# Extra Supplement—The Nursing Mirror

**Published:** 1889-08-10

**Authors:** 


### The Nursing Mirror.

*Extra Supplement.*

All Contributions for this Supplement should be addressed to the Editor, The Hospital, 139-140, Salisbury Court, Fleet Street, London, E.C., and should have the word "Nursing" plainly written in left-hand top corner of the envelope

### En Passant.

**Nurses as Witnesses.**—The case of Mrs. Maybrick, commonly called the Liverpool poisoning case, shows into what unexpected tragedies a nurse may suddenly step, and how necessary it is that she should be cautious in all her words and ways. Two Liverpool nurses were suddenly sent to attend on the late Mr. Maybrick. He was very ill at the time, and the nurses who were constantly with him had greater chances of observing his symptoms than anyone else. One point a nurse is always taught is to observe if sickness follows medicine or food, and in this case such observations were of the utmost value. Nothing which goes on in the sick-room should escape the nurse's observations, though we are far from asking her to draw deductions therefrom. "Hers not to reason why," but only to watch and report, and leave the doctor to do the reasoning.

**A Day's Pleasure.**—Many people have it in their power to do much good by slight effort and still less expense. Those who live in the country or near the sea during the summer might do great good by inviting, through the matron, a few members of the nursing staff of the hospital in their district for a day's outing. Those who have no such houses or grounds might send a small contribution to the matron to enable her to provide a day of pleasure for the staff. At Oldham this latter practice has been adopted with great success. Last week eight nurses from Oldham Infirmary went to Blackpool for a day's holiday, the remainder of the staff having previously visited the same place. The nurses left the institution at 8.30 a.m. and returned about 11 p.m., the expenses being paid out of monies sent to the matron, Miss Thompson, for the purpose. Miss Thompson has arranged to give a similar outing to the servants of the infirmary next week.

"**Recollections of a Nurse.**"—This is the title of a small book which we hope to review fully later on; at present we only desire to call attention to the fact that it was thought worthy of a leader in the *Daily News* of last Saturday. The writer of the leader says: "We are always reading diatribes and scarcasms against the present age—its want of faith, its want of hope, its want of charity, its selfishness, its levity, its morbid self-analysis, its inexhaustible craving for excitement. Yet the present age is the age of General Gordon and of Father Damien, and it is the age that brings out 'Recollections of a Nurse.' Not that our nurse in this particular instance did anything very wonderful, or passed through any very terrible experiences. She did what many women of her class and education have been doing every day this long time; and she does not make any fuss about it, any more than the other women do. That very fact is in itself, however, to the credit of her and of them, and of the age. Women—ladies, according to the conventional phrase—have gone back to the duties which ladies were proud to perform in the days of the Crusaders. Of course, we all know that before Florence Nightingale ever went to the wars, all manner of religious orders had been sending out their ministering sisters to look after the wounded on the battle-fields of the world. But it is something different, and perhaps still more encouraging to humanity, when women not pledged by any religious order to such deeds go out voluntarily to bear their part in relieving and redeeming the horrors of war."

**A Foreign Visitor.**—Many of our asylums have lately been visited by the Rev. Sister Therèse de Jésus, superioress of St. Jean de Dieu Asylum, at Quebec. We grumble somewhat at the scant attention and teaching given to most of our asylum attendants, and yet visitors constantly come from foreign parts to study our system. Sister Therèse returns to Canada shortly to put into practice what she has learnt during her travels.

**Brutality.**—There really is no other word to express our opinion of the rules for nurses in some hospitals. For instance, in one large hospital, where nurses are on duty from 7 a.m. to 9 p.m., with two hours off duty every other day, the nurses are forbidden to sit down while on duty. From twelve to fourteen hours of standing is enough to ruin any woman's health. Shopkeepers have been memorialised on this subject, but probably it never occurred to the public that those who are supposed to be learned in the laws of health should thus run contrary to all their knowledge. Another senseless and childish rule is to forbid all conversation at meals. A nurse's life is hard enough without such a useless restriction.

**Nursing the Insane.**—In a letter recently received from New South Wales, we learn that there is no accommodation there for the private treatment of the insane. Now cases of insanity amongst the wealthier classes are often hysteria or melancholia, and can best be treated by isolation. Every attendant knows that a lunatic who is constantly visited by friends and relations is far more difficult to manage than one who is left entirely under the doctor's and attendant's influence. A special nurse for each case can be provided at a cost of £4 a week; cases of delirium are generally charged £5 a week; this, of course, including board and residence. This is not much to pay if hope of cure can be held out by the isolation system. We wish some of our readers in asylums would be kind enough to give us their experiences; we specially wish to know whether nurses can manage a single patient or a group of patients best.

**Registration.**—At the risk of wearying our readers we must really quote part of a letter which lately appeared in the *Leeds Times* on this subject. Mr. Greeve-Fisher writes: "The letter of Dr. Allbutt in your last issue ought to have the hearty support of every true lover of liberty and progress. It is of the utmost importance that we should fully realise the dangers and the hindrance to intellectual and material advancement arising from the tyranny of various registration boards. There is no doubt that these are on the increase. Every trade, from town clerks to tooth-drawers and tinkers, must now have its union, its board, its council, its certificates of proficiency and its tyrannous monopoly. The legislation of professional jealousy is a danger to the public, and an injury even to the trades which rely upon this species of protection. There is, unfortunately, an inevitable evil attaching to this plausible word 'protection,' and we invariably find that when introduced in connection with trade or professions it means or involves 'restriction.' Fancied protection thus checks growth or expansion, and instead of the benefits expected simply produces injury. A fair field and no favour, free competition in the open market, equal liberty to all—these are the fundamental principles of prosperity and progress. No registrative monopoly can be arranged for the most intellectual trades. Every union has some log-rolling of its own to accomplish, and each knows that it can urge precedent in support of its own claims."

### Physiology for Nurses.

### The Hair and Nails.

The first essential for keeping the hair healt iy is to keep it clean, and to do this it is not necessary to use too much soap and water. Constant washing is not good for the hair, as it removes much of the natural grease and then dries up the hair; but when the hair has to be washed it is best to use good soap and yelk of egg, the egg being rubbed on the hair first and then a good lather of soap, and then all to be washed off with hot water. It is not a good plan to rub the hair violently with rough towels as it breaks the hairs very much, or even rubs them off. The best plan is, after washing off the lather with hot water, to pour some cold water over the head, and to rub the head gently with a towel, and then to allow it to dry naturally in a warm room. Long hair is not to be recommended; it is very tiring, and, except for naturally strong people, is very weakening, and the tight plaiting of it and its weight on the head has a very great tendency to give headache, not to speak of the painful dragging sensation of all the hairpins that are necessary to keep it in place. It is a good thing to cut the hair frequently to prevent the ends from splitting, but singeing is no good whatever; in fact, after what you now know about the injury done to the hair by allowing it to dry, you will at once see how hurtful it is to burn off the ends of the hair as well as drying up the rest of it with heat. The reason it is recommended is that people think a hair is a hollow tube and that after being cut it "bleeds," as it is called, which really only means that the fluid inside it runs out, but this is altogether a mistake. The hair is not a hollow tube, but is a tube filled with a material which sucks up moisture as a sponge might. Besides cleanliness, the hair also requires light and air—the air to carry off all the secretions from the skin, which if left would reach the hair itself, and, being left on the skin, would decompose and irritate it. Therefore, it is a great mistake to wear closely-made and heavy hats or handkerchiefs in which to wrap up the head. The hat or bonnet should be very light and porous and fit the head easily, and not be like a light band round it; and, as far as possible, hats and bonnets should be of straw. One reason why men get bald so early is on account of the hard hat they wear so many hours in the day, which lets in very little air to the head, and presses the head and forehead so much that it causes what is called congestion of the bloodvessels. You will notice there is always a fringe of hair below where the hat comes in bald people, which proves that it is the head covering which is at fault. Next, as regards hair dyes. It is very extraordinary how long ago it began to be the fashion to change the colour of the hair, and in those days nutgalls, pomegranate barks, and walnut shells, the ashes of plants and ivory black, were mixed with some kinds of animal fats and used. Long ago the ladies of Venice used to wear straw hats without any crown, and used to spread the hair it out loosely over the rim and expose it to the sun, and so they bleached it. Nowadays those to whom Nature has given dark hair and who wish to have it light, use what is called "golden fluid," which is really a sort of bleaching fluid. Most of these dyes contain lead, which is excessively poisonous; all the dyes are made of harmful substances, though perhaps all are not quite as bad as lead.

The nail, again, is only part of the cuticle; it is as if that special part of the cuticle were hardened and laid on the end of the finger; for the skin underneath is the cutis, and is commonly called the "quick"; the nail is thrown into ridges and furrows, and so also is the cutis beneath, and thus they fit in and are kept in place, and as the two are connected the nail is provided with nourishment. The finger nails grow much more quickly than those of the toes, and, moreover, the toe-nails are often hindered in their growth by short or narrow boots, and that is why so many people's toe-nails are of such a peculiar shape —long and narrow horns—or else they spread out too much at the sides and so form in-growing nails which are extremely painful. The skin which grows at the root of the nail and covers it often grows too much, and sometimes gets frayed and rough and cracked, which is very uncomfortable and even painful. In such cases it is not well to cut it with a knife or scissors, but after soaking the nails in hot water for a few minutes so as to soften it, it should be rubbed back with a towel and pressed down but not cut off. As far as possible the nails should be cleaned with hot water, soap and a nail-brush, and not scraped inside with a knife or pair of scissors which separates the nail from the skin underneath and makes a larger space for more dirt to gather next time, and it is almost impossible to help scratching the inside of the nail and making little grooves in which the dirt will stick and from which is will be difficult to dislodge it with a nail-brush.

*(To be continued.)*

### The Power of Laughter.

"Do 'ee listen to that!'

The speaker was a grey-headed old man—a bag of bones —every one of which ached its hardest from morning to night, and from night to morning, for there is no cure for "they rheumatics" in this world. He was sitting up in bed, huddling his coloured jacket round him with many a shiver, and listening eagerly to a sweet ringing sound that came at intervals from a cot a good way down the opposite side of the ward.

"It be a rare treat, bain't it?" said his neighbour in the next bed.

And it was a treat; there's no doubt about that. A child's laugh! Can human ears imagine sweeter music, so full of pure, unrestrained, joylike echoes from a better, brighter world than ours?

"What do'ee suppose is the matter with he that he should be in 'orspittle?" asked Jeremiah Brown, rubbing his rheumatic elbow.

"Heard tell 'twas a habscess in his ankle, pore little chap; but he *is* full of life. and that gay! Nobody'd think as *he* ailed aught, would they now, for sure?" and the two old men pondered how and why a body should laugh over aches and pains, instead of groaning and grumbling—as they did.

"Johnny, you're just like the canary in my room," said the cheery nurse, who was doing something to the ankle of the little chap who could be "that gay" in a hospital. "It sings its heart out, and fairly deaves me in the mornings, and you laugh and chuckle much in the same way. You ought to be in the canary's cage; you would be a lively pair, chirruping and laughing all day long. Really, you should have been a lark; perhaps you were long ago, before you became a little boy, who knows!" An idea which struck Johnny as being so particularly funny that a louder, sweeter peal of laughter than ever made everyone in the ward stretch their heads from their pillows to find out what could be the joke—for joke it must be.

"What is that? Who can possibly be laughing like that *here*?" asked a lady—an elderly lady with melancholy eyes and a face all the lines of which drooped down instead of upwards. Think, for a moment, what a world of difference lies in that distinction! She was being conducted over the ward by the lady superintendent, and from various signs must have been considered a very distinguished visitor. So she was, in a sense, for Miss Orme suffered from an embarrassment of riches as much as did that celebrated old woman who lived in a shoe, only in Miss Orme's case the riches were tucked away in banks, in Government stocks, and all that sort of thing. She was not any the happier, poor lady, for it all. One cannot eat gold, it is very indigestible and unsatisfying. So Miss Orme was rather starved, so to speak, and her friends whispered, shaking their melancholy heads: "Dear, dear, what a pity. Not a chick nor a child to leave it all to!"

"I think I should like to see that laugher," said Miss Orme curiously, and much as if she expected to see a chimpanzee or a similar monster.

"Oh, certainly," answered the lady superintendent, amiably; "it is little Johnny, the pet of the ward at present, whom no poverty, no loneliness, no sickness, even, can daunt. He was brought here in a pitiable state, but we never hear a murmur nor a cry from the fearless little creature. Poor child, I'm afraid the cruel, hard life before him will quench all that bubbling happiness, though. See, here he is!"

Miss Orme gazed down stolidly at the smiling morsel of humanity who could not be crushed, and Johnny stared up at her out of grey twinkling eyes set round with inky black lashes—eyes that stirred an old memory in Miss Orme's heart—a memory she had thought to be dead and gone. In her far-away girlhood she had known and loved such eyes; loved them, but had not scrupled to quench their lovelight by the cruel suspicions born of that miserable burden of gold which had warped her woman's nature. There was a strange, long silence; the superintendent fidgetted, nurses passing to and fro gazed and wondered, but no one ventured to interrupt it.

"Did you say this child is an orphan? When he gets well, let me know; I want him." The words were terse and abruptly spoken; all Miss Orme's speeches were so, but they were never questioned.

Down in the South, in a lovely, quiet, peaceful seaside place, Miss Orme is, at present, busily occupied—unusually so. She has caught her human lark—the pet of the ward—and, for the first time, is tasting the joy of living for another, instead of for herself. People comment—as people will—and marvel. But the once lonely rich woman cares not; she is happy at last, as she may well be, for human "hobbies" are by a long way the most satisfying in the end.

### A Probationer's Diary.

Sunday, October 14.—No serious cases; so at 10 a.m. the two wards were left to me, only the house surgeon came round; at one I was off duty, dressed, and went to Birmingham. I arrived at hospital at night (9.40) tired. Hospital work seems a true type of a mortal mind, "ever revolving."

*Monday, October 15th.*—Lovely autumn morning. One new patient with a bad ankle, which was lanced, after a warm bath: put her to bed. After 5 p.m. I went to the wards opposite, women's medical, 24 beds. A bad case of puerperal fever, and one poor thing—dying from Bright's disease—has had to-day two attacks of ureamic convulsions, alleviated by giving hypodermic injections of chloroform or ether. Some few are convalescent, and one very useful and anxious to assist. At 7.30 the majority of probationers went to the Bell Library to a lecture by the house surgeon on the structure of bones.

*Tuesday, October 16th.*—Three new patients: first, typhoid, a girl about 18; 2nd, exema, not so bad; and 3rd, pneumonia, temperature 101. The poor woman with Brights had no more convulsions, but died this morning; her sister was with her. I was off duty, 2 till 4, fearful headache. The chaplain came and read prayers in the long ward. The charge nurse, wrote the report, and altered the diet-sheet at supper. I heard a man had been brought into the accident ward, his lower limbs crushed, buried 48 hours in a coal mine; it is yet uncertain if both his legs will not have to be amputated, as they cannot restore the suspended circulation.

*Wednesday, October 17th.*—On duty as usual at 7, one pneumonia case, very bad; expected hœmorrhage from typhoid; puerperal fever, better; other cases mending. Visitors from 2 till 4, raining heavily. The ward maids don't care for wet visiting afternoons, as the wet plays sad havoc with the polished boards.

*Thursday, 18th.*—A lovely morning dawned in spite of yesterday's wet. Typhoid case stationary, no hœmorrhage; pneumonia very bad. I sent my child a pair of socks one of the poor women knitted. The doctors did not come round till one. Two fresh patients, both heart disease. A convalescent from rheumatic fever left us to-day. How tired one gets as the day closes! I think the polished floors have a great deal to do with feet aching.

*Friday, 19th.*—Our pneumonia slightly better; temperature lower. Three fresh cases, one a girl with chorea; it was hard to give her a bath; a spasm coming on when I gave her medicine, she bit a large piece out of glass, which luckily dropped on bed. Two patients gone out. Puerperal fever case still mending.

*Saturday, 20th.*—I hear from downstairs a patient is just removed from the burnt ward (having developed smallpox) to the fever block. I joined a fellow probationer, from four till six, for a walk; had to give in at seven and go to bed, as I had a sick headache.

*Sunday, 21st.*—The charge nurse off duty. A probationer, longer trained than I, took charge. All our cases mending except one bad heart case; poor woman, she is angry the doctor refuses to allow her vegetables. Visitors come from two till three. I took the checks. I feel better to-day; off duty from 6.30.

*Monday, 22nd.*—No fresh cases; all mending except the chorea and heart case; the latter is very sad, so much dropsy. I went to 18 ward; nothing very serious; several gone out since I left. Lecture at night in the Bell library, principally about the "facia," or sheath encasing the muscles, illustrated by the leg which was amputated from the poor fellow in the accident ward. Every means had proved futile to restore the circulation in it.

*Tuesday, 23rd.*—Our nurse gone home to day for her health. I was on duty by myself till the night nurse came. The house surgeon came up early for the diet-sheet.

*Wednesday, 24th.*—A very busy day. Even now I can recall the face of the honorary surgeon, as he said to one patient, a woman of about fifty-five, "Well, ma'am, we think an operation necessary; it is life or death, a few die, many recover." The poor woman, standing, so it seemed, on a precipice, replied, "I am ready, sir, for you to do as you think fit." So this day the operation was at 11.30. The assistant house surgeon gave the anæsthetic, and a large ovarian tumour was removed, containing close on two gallons of fluid. The tumour removed looked like a large pig's bladder, only thicker, with sundry smaller growths upon it. The poor woman seemed comfortable. I did not get off duty till night; had my usual hot bath, but could not sleep, my head aching like mad. The night nurse came up at two with a message, if I was awake, and as I was and fearfully sick, fetched me a cup of tea, after which I slept till morning.

*Thursday, 25th.*—I fancy it was the chloroform upset me; yes, the smell has always been most nauseous since I had it a few years back. I feel well to-day. Went through the usual probationer's routine; all rush till 9.30, the patients' lunch. No fresh cases, the "ovarian" satisfactory, is having 1oz. of ice and milk in the hour; complains only of thirst. I sat with her in the evening till the night nurse came at nine.

### Everybody's Opinion.

*[Correspondence on all subjects is invited, but we cannot in any way be responsible for the opinions expressed by our correspondents. No communications can be entertained if the name and address of the correspondent is not given, or unless one side of the paper only be written on.]*

### REGISTRATION.

NURSE J. C. writes: If "Courage" had made any enquiry into the rules of the B.N.A. she would have found that no person could be admitted without testimonials from doctors, stating her qualifications as a nurse. I do not think doctors give these to wardmaids and scrubbers even if they possess half-a-crown.

### Appointments.

[It is requested that successful candidates will send a copy of their applications and testimonials, with date of election, to THE EDITOR, The Lodge, Porchester-square, W.]

ALFRED HOSPITAL, MELBOURNE.—Out of 28 applicants for the post of assistant matron, at a salary of £75 per annum, Mrs. C. Unger was elected the successful candidate on June 6th.

LONDON HOSPITAL.—The value of passing well at examinations is shown in the case of Miss A. L. Ridal, who lately took second prize, and has now been appointed Sister Cotton.

MANCHESTER ROYAL EYE HOSPITAL.—Miss Marian Violet Black, formerly matron of Atkinson Morley's Convalescent Hospital, Wimbledon, has been appointed matron of this hospital.

### Vacancies.

The following vacancies are announced:

**Matron.**

Kensington Infirmary, £100.

**Sister.**

Monsall Fever Hospital, £30.

**Nurses.**

Barrow-in-Furness Hospital, £20.

Birmingham Fever Hospital.

Birmingham Orthopædic Hospital.

Brompton Consumption Hospital.

Civil Hospital, Gibraltar (two), £25.

Edinburgh Jubilee Institute.

Guernsey Cottage Hospital, £25.

Halifax Fever Hospital (assistant), £18.

Hampshire Nurses' Institute.

Holborn Union (night), £20.

Jersey Nurses' Institute (two), £25.

Kendal Hospital (night), £28.

Kingston Nurses' Home, Hull.

Maidstone Hospital, £26.

Manchester Consumptive Hospital, £20.

Miss Stewart's Institute, Glasgow.

National Hospital, Queen-square, £25.

Rochdale Infirmary, £22.

39, Royal-avenue, Chelsea.

Sauchiehall-street, Glasgow, £25.

Stratford-on-Avon Hospital, £25.

St. Saviour's Infirmary, £20.

Ventnor Nursing Institution (several).

Victoria Home for Nurses, Bournemouth (several).

West London Hospital, £24.

West Malling Institution.

Weston-Super-Mare Nurses' Institute (several).

Wilts County Asylum, £18.

Wolverhampton Nursing Institution.

**District Nurses.**

Bristol.
Kingston Nursing Association, £70

**Probationers.**

Barnet Cottage Hospital.

Barrow-in-Furness Hospital, £16.

Carmarthen Infirmary, £8.

Coldstream Cottage Hospital.

Gt. Yarmouth Hospital (two), £20.

Home for Invalids, Birkenhead.

Manchester Consumptive Hospital

Wolverhampton Nursing Institution.

### Winter Amusements and Remunerative Work.

### AN EXHIBITION OF DOLL NURSES.

THE EDITOR of THE HOSPITAL has much pleasure in offering to nurses prizes to the value of £10 for the three best dressed sets of dolls representing the nursing staff of any hospital. The dolls must not be shorter than four inches nor taller than twelve inches, and must represent the sister, charge-nurse, and probationer of the hospital. Any nurse intending to compete must send her name and address on a post-card, headed "Doll Competition," together with the name of the institution or hospital, the uniform of which is to be faithfully reproduced, to the Editor of THE HOSPITAL, The Lodge, Porchester-square, London, W. *before September 1.* Further particulars will be announced later on, and arrangements will be made for a public exhibition of the dolls where nurses will be able to see the show. The number and amount of the prizes will not be fixed at present in deference to a general wish, and suggestions on this point are invited.

### Examination Questions.

(The Editor will be glad of specimens of similar questions.)

1. To what special points should you pay attention in nursing a case of typhoid fever, scarlet fever, rheumatic fever?
2. Give a short account of the circulation of the blood, and explain any special points to be attended to in the nursing of cases of heart disease?
3. What preparations should a nurse make (in a private house) for the following operations, and what should be her principal care during the first 24 hours after each operation? Ovariotomy, hare-lip, amputation of thigh, lithotomy?
4. What precaution should you take to prevent the occurrence of a bed-sore? and how should you treat one when existing?
5. Describe in detail any form of "Artificial Respiration," explaining any points you consider important.
6. State what you know of (1) the structure of bone, (2) the course of cancer, (3) the phenomena of bleeding, (4) the antiseptic treatment of wounds.

### Notes and Queries.

TO CORRESPONDENTS.—1. Questions may be written on post-cards. 2. Advertisements in disguise are inadmissible. 3. In answering a query please quote the number. 4. If a private answer is desired, a stamped addressed envelope must be forwarded.

**Queries.**

(61) *Probationers.*—Please give me the names of some hospitals where I could be received as a probationer for a short time. *Minnie.*

(62) *Obstetric Nursing.*—What books are likely to be useful to one learning obstetric nursing? *Nurse Rose.*

(63) *Titled Nurses.*—Do ladies of title ever take up nursing? *Gwen.*

(64) *Training Schools.*—Please tell me where I can be trained as a nurse free of charge. *A Would-be Nurse.*

(65) *A Book for the Pocket.*—Where can I obtain Mr. Walter Pye's book on "Elementary Bandaging," mentioned last week? *Isleworth.*

**Answers.**

(59) If district nursing would suit the requirements of "A. H.," will she kindly communicate with Sister Katherine, St. Mary's Mission House, Plaistow, E.

(61) *Probationers.*—St. Mary's Hospital, Paddington; The London Hospital, Whitechapel; Sussex County Hospital, Brighton; in fact, nearly every hospital takes probationers for from three months to a year, on payment of a guinea a week.

(62) *Obstetric Nursing.*—"Obstetric Aphorisms," by Swayne; "Diseases of Women," McNaughten Jones; and "Obstetric Hints," by Coffin.

(63) *Titled Nurses.*—Lady Amberley and the Baroness Ebba Bostron have nursed at St. Mary's; Lady Colin Campbell wore cap and apron for a short time at St. George's; one of the Queen of Sweden's retinue spent some time at The London; but we never heard of a titled nurse (unless a member of a sisterhood), who stuck to the work.

(64) *Training Schools.*—Any hospital will grant you training in return for either two or three years' service. Apply at the hospital nearest to you and ask to see the matron.

(65) *A Book for the Pocket.*—From Messrs. Trubner and Co., 57, Ludgate-hill, E.C., or through any bookseller.

*Home for Epileptic Girl.*—*E. A. H.* must send her name, as no attention can be paid to letters which do not contain the full name and address of the correspondent from whom they emanate.

